# Rib biomechanical properties exhibit diagnostic potential for accurate ageing in forensic investigations

**DOI:** 10.1371/journal.pone.0176785

**Published:** 2017-05-17

**Authors:** Andrea Bonicelli, Bledar Xhemali, Elena F. Kranioti, Peter Zioupos

**Affiliations:** 1 Edinburgh Unit for Forensic Anthropology, School of History Classics and Archaeology, University of Edinburgh, Edinburgh, United Kingdom; 2 Musculoskeletal & Medicolegal Research Group, Cranfield Forensic Institute, Defence Academy of the UK, Shrivenham, United Kingdom; 3 Forensic Institute, Department of Forensic Medicine, Tirana, Albania; 4 Department of Forensic Sciences, Faculty of Medicine, University of Crete, Heraklion, Crete, Greece; University of Sheffield, UNITED KINGDOM

## Abstract

Age estimation remains one of the most challenging tasks in forensic practice when establishing a biological profile of unknown skeletonised remains. Morphological methods based on developmental markers of bones can provide accurate age estimates at a young age, but become highly unreliable for ages over 35 when all developmental markers disappear. This study explores the changes in the biomechanical properties of bone tissue and matrix, which continue to change with age even after skeletal maturity, and their potential value for age estimation. As a proof of concept we investigated the relationship of 28 variables at the macroscopic and microscopic level in rib autopsy samples from 24 individuals. Stepwise regression analysis produced a number of equations one of which with seven variables showed an R^2^ = 0.949; a mean residual error of 2.13 yrs ±0.4 (SD) and a maximum residual error value of 2.88 yrs. For forensic purposes, by using only bench top machines in tests which can be carried out within 36 hrs, a set of just 3 variables produced an equation with an R^2^ = 0.902 a mean residual error of 3.38 yrs ±2.6 (SD) and a maximum observed residual error 9.26yrs. This method outstrips all existing age-at-death methods based on ribs, thus providing a novel lab based accurate tool in the forensic investigation of human remains. The present application is optimised for fresh (uncompromised by taphonomic conditions) remains, but the potential of the principle and method is vast once the trends of the biomechanical variables are established for other environmental conditions and circumstances.

## Introduction

Age-at-death estimation remains one of the most challenging tasks in forensic practice when establishing a biological profile from unknown heavily fragmented or skeletonised human remains. The methodological choice is subject to the general pattern of preservation of the remains and the specific nature of the case[1].

During childhood, morphological methods based on developmental traits of bone can provide extremely accurate results, but taphonomic changes increase the difficulty of the procedure[1–3]. In adulthood, morphological methods, although easily applicable, are often inaccurate for ages over 35 years old when all developmental markers disappear and thus cannot be accepted by the legal system. These facts have led to the development of methods based on the quantifiable degeneration of bone[1–8]. Furthermore, each method is highly population and sex specific[1] and individual differences must be considered when interpreting the results[9].

Garvin and Passalacqua[3] compared three of the most commonly used morphological age estimation methods and studied the effect of the level of experience of different operators on the application of the methods and the final age estimates. The results show that the inter-observer bias in the methodological application and consequent age estimation are not predictable, which makes the application of such methods in a forensic situation problematic.

When the skeleton is preserved intact, a number of methods based on the pelvis, skull, rib cage, and dentition can be applied and the final age estimate will be based on the combination of these age estimates. In numerous occasions, though, the remains are only partially retrieved and several important age estimation markers may be missing. This is one of the reasons that numerous age estimation techniques were developed on ribs, a small skeletal element that can be easily obtained and examined during a forensic examination. For instance, Işcan et al.[8] developed a method based on the qualitative observation of metamorphic changes of the sternal end of the fourth rib caused by a progressive age-related ossification of the cartilage tissue connecting the ribs to the sternum. It has been proven to be generally very accurate (95% of accurate estimation) and suitable for different populations[1]. However, this method, as well as other morphological methods, has a high level of inter-observer error and a decreased accuracy when aging older individuals[8].

The rib was also employed in histomorphometric studies based on bone remodelling[4–5]. The original method by Stout and Paine[4] on the sixth rib gave extremely accurate results especially when combined with the clavicle. What is highlighted by the authors themselves is the fact that, although the estimation has a 95% of accuracy, the method fails to give a reliable age prediction for individuals over 40 years-old due to the overlapping of secondary osteons[4]. Moreover, this method underestimates age significantly when applied to other populations[10]. Cho *et al*. [11] have provided an updated histomorphometric method which employs several additional parameters and seems to account for older ages and ethnicity. This method was tested using a large sample (N = 213) from South Africa and reported 6–11% of the sample falling out of the 95% confidence prediction range (+/-24.4 years) of the original study[12]. These authors favour the use of the unknown-ethnicity formula and reject the hypothesis that population specific-equations are needed, since equations based on their sample did not perform any better than Cho’s equations. This paper is one of the few validation studies with such a large sample and contains criticisms as to the value of regression in the analysis of histomorphometric variables.

Other laboratory-based methods include the aspartic acid racemization techniques that are based on the heat-dependent gradual transformation of specific proteins during life[13–16]. Several studies confirm the high accuracy of such techniques, however, they have significant limitations: high demand in time, equipment and expertise, poor results for particular categories, such as mature females and are ineffective for post-mortem interval of more than 20 years[1,13,17,18].

Radiocarbon dating methods use the variation of atmospheric ^14^C levels, which are ultimately incorporated into living tissues to date the formation of proteins in the lens, brain neurons and bone. The dramatic increase of the amount of atmospheric ^14^C from 1955 to 1963, due to nuclear bomb testing allows for the accurate estimation of the time that tissues with slow ^14^C turnover were formed, thus producing an accurate estimation of the date of birth. Alkass et al.[19] used dental enamel to combine aspartic acid racemization and radiocarbon dating techniques on a known age sample. According to the results, radiocarbon analysis showed an overall absolute error of 1.0 +/- 0.6 years while aspartic acid racemization showed an overall absolute error of 5.4 +/- 4.2 years.

DNA methylation, one well-known epigenetic modification, has also been shown to correlate with age[20,21]. More specifically, the global level of methylated genomic DNA decreases with increased age[20]. A recent study on Chinese Han female monozygotic twins identified 2,957 novel age-associated DNA methylation sites (P<0.01 and R^2^>0.5) in blood. Eleven CpG sites were used to develop an age regression model which exhibited a mean absolute deviation from real chronological age of 2.8 years and an average accuracy of 4.7 years[21]. It must be stressed though that population, sex and environmental exposure (e.g. smoking, alcohol consumption) influences DNA methylation[21].

In addition to the existing methodologic analytical methods mentioned above, Zioupos et al.[18] studied numerous physical characteristics of femoral bone at the macro- and microscopic level and proposed a method that could approximate age with accuracy of +/-1 year. These characteristics are based on changes in the biomechanical properties of bone and the properties of the bone matrix, which change with age even after skeletal maturity, and include traceable features such as the wet and dry apparent density, porosity, organic/mineral/water fractions, collagen thermal degradation and the osteonal and matrix micro-hardness[22–23]. The authors offered several alternative procedures as well as various combinations of variables in order to present an accurate method that was versatile enough to be carried out in less than 24 hours, or without the need for expensive equipment. The current paper presents a study that follows the same methodological approach [18] for a larger sample of ribs. The underlying hypothesis is that a methodology based on biomechanical properties and biomechanically related structural characteristics may be more widely applicable and for more skeletal sites. An alternative pertinent site for forensics is the rib and for that a different set of biomechanical variables and parameters are needed that suit rib anatomy and physiology. Physical characteristics of ribs are less influenced by mechanical stress compared to the femur throughout life[24–25], but more influenced by hormonal and metabolic changes.

After the complete maturation of the skeletal system, the process of remodelling maintains the structural integrity of the bone, which is constantly subjected to mechanical stress. According to Martin[26], cyclic loading is the main cause for remodelling. When remodelling does not succeed in maintaining the integrity of the bone then, for excessive loads or pathological conditions, there is an accumulation of visible microdamage on the bone surface that results in a deterioration of mechanical properties[27–28]. Ageing causes local hypermineralisation patterns, not dissimilar to bio-mineralisation patterns seen in some extreme biological examples[29], where the material becomes extremely brittle. In–vivo fatigue microcracks have been seen to accumulate in such hypermineralised areas. The accumulation of microdamage is related to cyclic loading, and previous studies have related this phenomenon to physiological aging[22,30–32] and in ribs in particular[33]. Furthermore, collagen, the main component of the organic matrix in bone, has been found to play a key part in maintaining the toughness and the structural integrity of bone and its deterioration has been repeatedly demonstrated with age[34–36]. Compositional and structural properties of the mineral matrix are also affected, resulting too in a decrease of the overall mechanical integrity of the tissue[36]. Lastly, ribs are convenient to access from the thoracic cage during autopsy, which would increase the applicability of the method.

This project, therefore, focuses on the analysis of the rib bone matrix to propose a laboratory-based method that optimises time and resources to produce an accurate age estimation method that can be easily replicated without any forensic expertise. The small sample suggests the potential of this type of analysis in predicting age at death but does not guarantee that the method would not be affected by other factors as further analysed in the discussion. Due to the small sample size, this study should be treated as a proof of concept, and upon positive results, a follow-up study with adequate sample size and reduced biases would be designed and conducted.

## Material & methods

### Study sample

This study used autopsy material (N = 24) from two forensic departments in Albania and Greece (Table 1 for details). The sample was divided into two sets of twelve 4th ribs each. The Greek sample derived from the Dept of Forensic Sciences of the University of Crete and was composed of 10 males (age 20–68; mean = 41.1/-17.1 yrs) and 2 females (age 22,40; mean = 31/-12.7 yrs). The Albanian specimens came from the Forensic Institute of the Ministry of Justice in Tirana, Albania and consisted of 8 males (age 30–57; mean = 41.3 +/-10.7 yrs) and 4 females (age 29–58; mean = 45/-13.4 yrs).

**Table 1:** Description of the sample employed in the study (1 = male and 2 = female).

Code	Sex	Age	Pathology	Cause of death
A1	1	45	NO Pathology	Self-poisoning
A2	2	39	Generalised atherosclerosis	sudden death
A6	1	38	NO Pathology	Traffic accident
A7	2	58	NO Pathology	Traffic accident
A8	1	58	Coronary atherosclerosis, Valvular hypertension	Sudden death
A9	1	30	NO Pathology	Mechanical asphyxia
A11	2	29	NO Pathology	Gunshot wound
A12	1	48	NO Pathology	Asphyxia
A13	1	57	Coronary atherosclerosis	Sudden death
A17	2	54	Myocarditis, pulmonary oedema	Sudden death
A19	1	44	NO Pathology	Asphyxia
A20	1	30	NO Pathology	Gunshot wound
C2	1	68	Hypertension, Coronary atherosclerosis,Progressive Supranuclear Palsy	Gunshot wound
C3	1	28	NO Pathology	Traffic accident
C4	1	40	NO Pathology	Traffic accident
C5	2	22	NO Pathology	Traffic accident
C6	2	40	NO Pathology	Traffic accident
C7	1	23	NO Pathology	Traffic accident
C8	1	20	NO Pathology	Asphyxia
C9	1	62	Alcohol abuse, smoking	Sudden death
C10	1	47	NO Pathology	Asphyxia
C12	1	48	Fatty liver	Myocardial infarction
C13	1	52	Hypertension	Sudden death
C22	1	23	NO Pathology	Traffic accident
Mean age	41.8			
SD	13.9			

### Ethical approvals and permits

The study protocol was approved by the Ethics Committee of the School of History, Classics and Archaeology of the University of Edinburgh (Ethics Assessment Level 2), the Ethics Committee of the University Hospital of Heraklion, Crete, Greece (Protocol Number 530) and the General Prosecution Office of the Ministry of Justice (Protocol Number 1797/3 A. Xh.), and the Institute of Forensic Medicine (Protocol Number 795) in Tirana, Albania. The study used fragments of ribs from routine autopsies of individuals for whom their next of kin signed an informed consent form or cases of unidentified remains for which rib sampling was conducted for diagnostic purposes using standard histomorphometric techniques with permits from the relevant judicial authorities. All methods were carried out in accordance with the approved guidelines and the appropriate standards applying in the medicolegal context.

### Sample preparation

The samples were shipped in a polyesterene box with dry ice in order to preserve their native condition[37]. Each was ~5 cm in length and was taken from the straightest portion of the shaft. For the entire preparation and experimental examination the specimens were stored in labelled airtight plastic bags at -20°C. The soft tissue on the bone was precisely removed using a disposable surgical knife and making sure to completely eliminate the periosteum without compromising the integrity of the. A thin transverse session (~5 mm) from each specimen was obtained using a Struers® Accutom wafering saw equipped with diamond impregnated blade (300 μm) and was cooled down using deionised water. The same machine was employed to divide the rest of the bone into two halves. When curvature or thickness did not allow for the procedure to be completed as stated, a Dremel® 3000 drill equipped with an abrasive cutting disk was used under continuous irrigation with deionised water in order to produce the thin section.

### Staining

The transverse sections were stained in plastic vials on a spinning mixer with a solution of Basic Fuchsin and Ethanol 70% for 14 days, with the solution replaced after seven days. The rest of the tissue was immersed in two baths of Ethanol 100% and 50%, respectively for three hours and then was left spinning overnight in deionised water. The stained sections were dried completely at room temperature (~12 hrs) and then embedded in epoxy resin (Metprep Kleer-Set Type SSS) to make the histological surface visible. After 24 hours the resin blocks were metallographically polished using an automatic Struers RotoPol-15 with 203 mm silicon carbide abrasive disks grinding paper of decreasing grit size (400, 800, 1200, 2500) on a MasterTex cloth with Alumina 3B 6OZ.

### Nanoindentation

**Fig 1:**
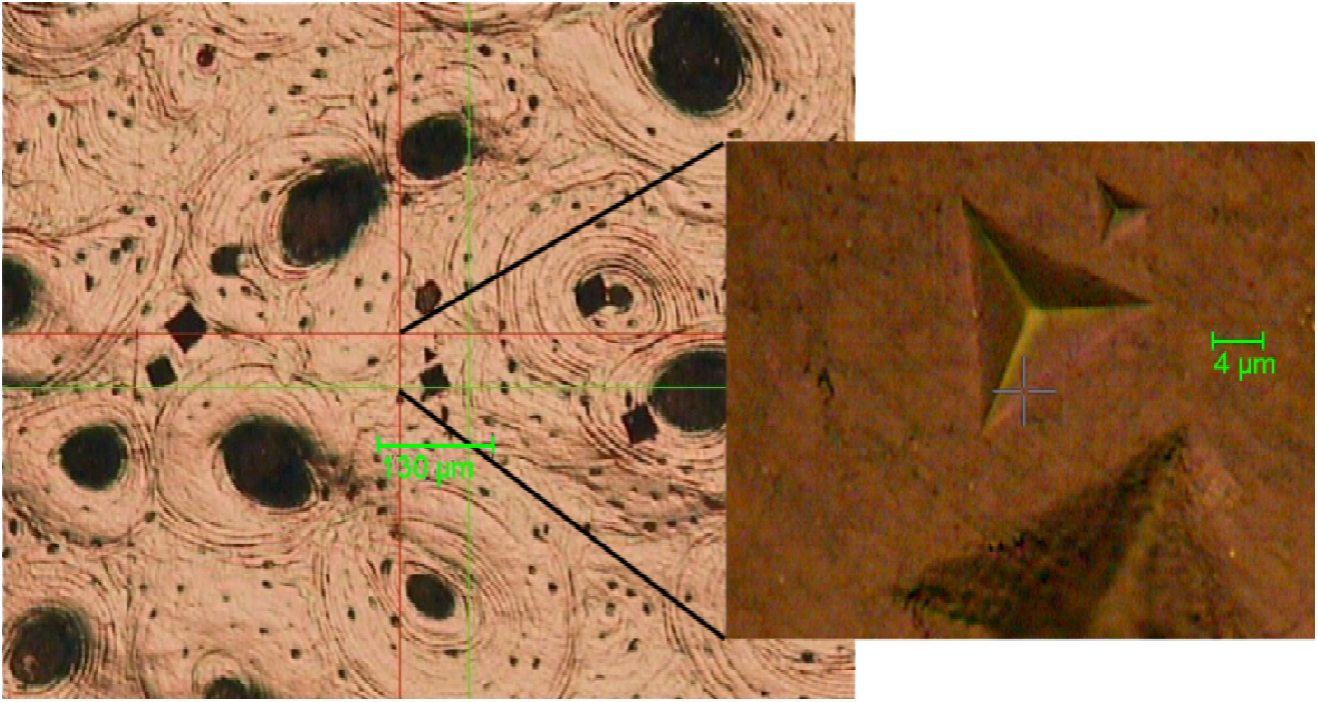
Detail of cortical bone matrix showing micro- and nano-indentations in an interstitial bone area.

Nanoindentation was performed using a CSM-NHT (system v.3.75, CSM, 2034 Peseux, Switzerland) instrument, at 10 mN maximum hold load (20 mN/min loading/unloading speed) with 30s long load/hold/unload stages. For each specimen, six indentations were performed on each of three different secondary osteons and in three surrounding interstitial bone areas nearby (see Fig 1). Each osteon was chosen from different sectors of the bone (pleural surface, cutaneous surface and one of the two edges chosen according with the regularity of the surface). Universal Hardness (H in MPa) was calculated from load and contact area, Elastic Modulus (E_IT_ in GPa) was obtained (assuming a Poisson’s ratio value of ν = 0.3) in the unloading phase as per the Oliver and Pharr method[38]. Indentation creep (C_IT_ in %) was calculated by the proportional increase in depth occurring while the load is held at its maximum level (for 30s) and its measurement reflects the viscoelasticity of the tissue. The elastic portion (n_IT_ in %) of the indentation work was obtained by examining the percentage ratio of the elastically recovered energy over the total energy (elastic + plastic) input during performing an indentation sequence. During the test, attention was taken in avoiding factors that create ‘experimental noise’, such as levelling the sample to allow the indenter to penetrate at right angles, minimising the external vibrations and discarding any asymmetric or problematic readings[39]. An INDENTEC HWDM-7 instrument, equipped with a square-shaped pyramid diamond tip of θ = 136°, was employed to produce Vickers microhardness (HV in Vickers) values for osteonal and interstitial bone areas for each specimen. The maximum load in these tests was set at 50 mN.

### Porosity

**Fig 2:**
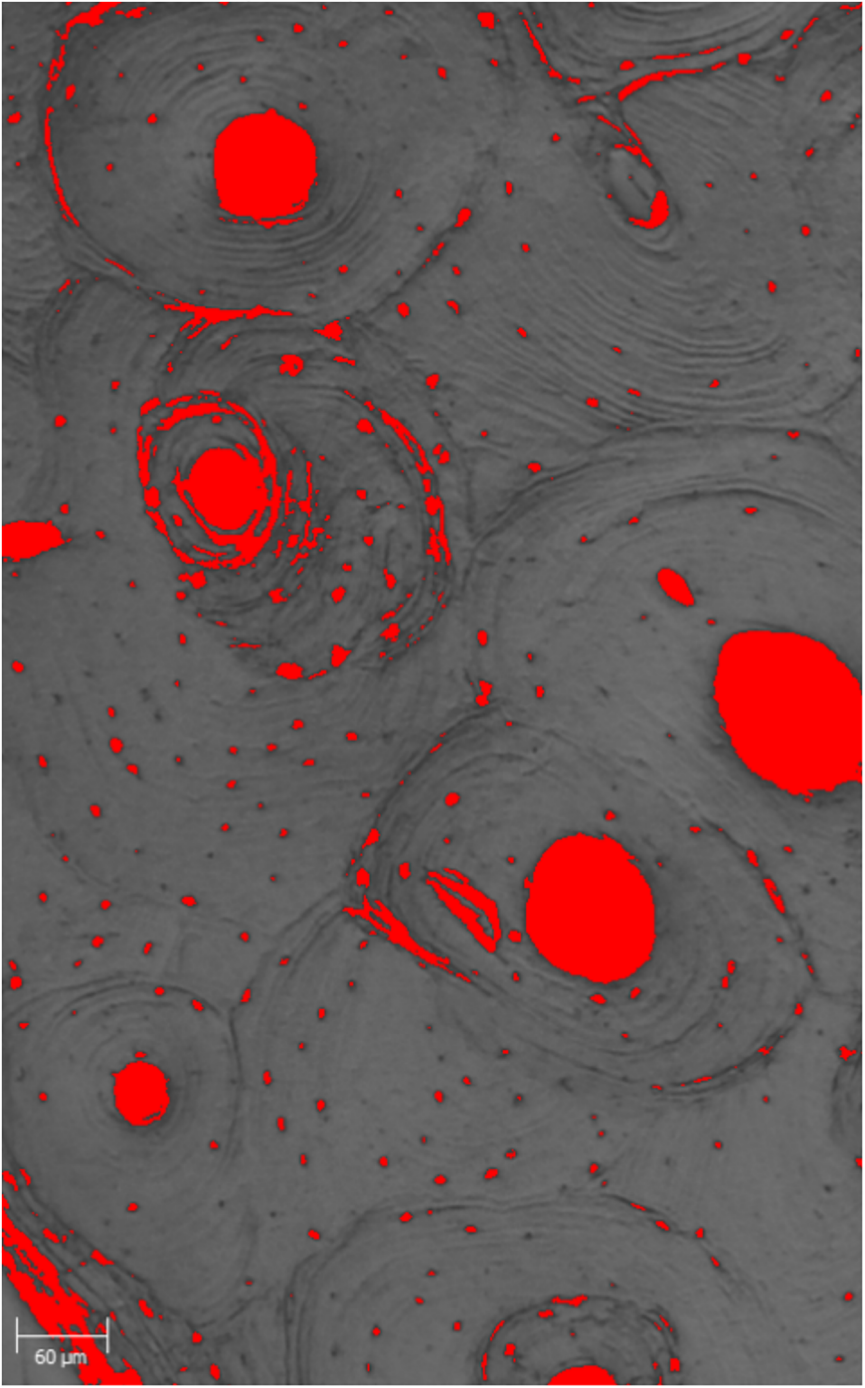
Pictures of a cortical bone section after conversion into 16-bit and the application of the threshold mask with ImageJ.

Optical porosity (%Po.Ar in %) was obtained from three pictures (respectively from cutaneous surface, pleural surface and from one of the two edges) taken for each specimen with a confocal transmitted light microscope 50× and the use of ImageJ RBS. The picture was cropped to select areas completely occupied by bone, then converted into 16-bit and a threshold mask was applied to highlight the voids in the tissue (Fig 2). If any of the osteonal canals was not stained properly a correction was made manually. The volume fraction was calculated using the open source software BoneJ and was then transformed into a percentage value. The three values for each individual were averaged in order to obtain a unique measurement for porosity. Osteocytic lacunae were included when automatically selected by the software.

### Microdamage

**Fig 3:**
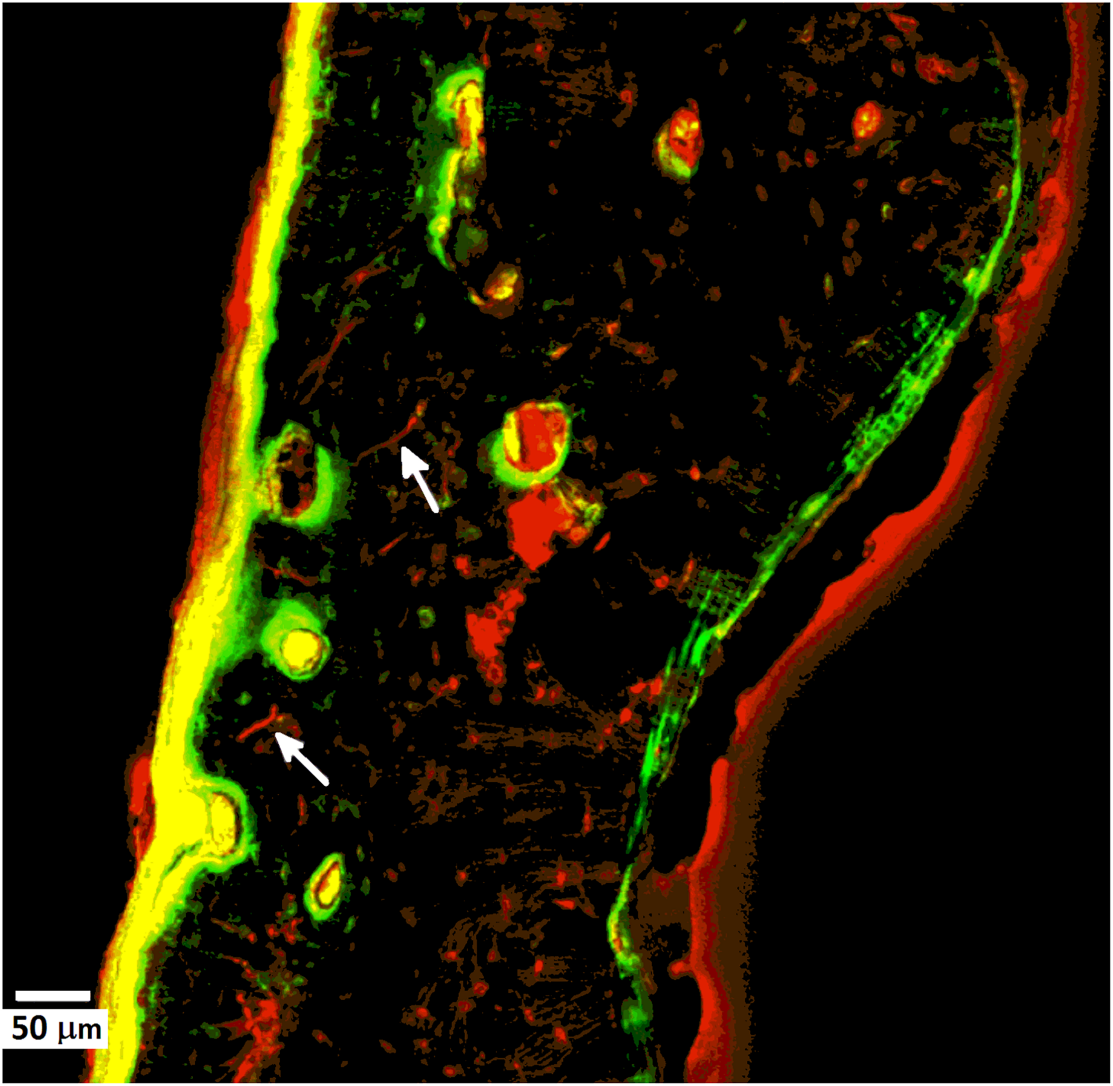
Examples of verified in-vivo damage micro-cracks (white arrows) visualised in a fluorescence microscope.

A NIKON A1R with a 10× Plan Fluor/NA 0.3 objective was used to produce the numerical density (Cr.Dn in n°/mm^2^) and surface density (Cr.S.Dn in mm/mm^2^) of in-vivo microcracks. Three pictures were taken on the cutaneous surface, pleural surface and on one of the two edges. For the numerical density, the area of the bone was calculated without removing the porosity. Cracks were identified and counted for the three areas added up and the value divided by the total area inspected. The same procedure was followed for surface density with the exception that length of each crack was recorded in order to calculate the total length and the value was divided by the total surface area of bone examined. In-vivo microcracks were labelled using basic Fuchsin fluorescent sodium salt (in water) solutions. In order to accurately distinguish between genuine cracks and artefacts, the following features were deemed necessary to be present: (1) they must have sharp edges and must be stained all around their length and width; (2) changing the depth focus the halo of Fuchsin and Fluorescein dyes must penetrate the depth of the crack under visible light (predominantly for Fuchsin) and UV light for Fluorescein; (3) genuine damage cracks ought to be larger than canaliculi but smaller than the vascular canal[30,32]. Consequently, cracks stained and verified by both Fuchsin and Fluorescein dyes (Fig 3) were counted and measured. Quantitative measurements were taken using Fiji open source software.

### Density determination

Rib fragments (~20 mg) were dried off completely at room temperature for 24 hours and then tested using a helium pycnometer (Mycrometric Accupyc 1330). Their weight was recorded using an electronic balance (Mettler Toledo® College B154) in order to obtain the first density measurement (Dn_pyc_ in g/cm^3^).

### Thermal stability of bone collagen

The remaining bone was dried out completely at room temperature and in order to powder it was processed using a Retsch Mixer miller 2000 by cycling for 1 minute and at 60 Hz. In between the two different cycles the powder was filtered using a 106 μm sieve in order to obtain a fine and homogeneous sample. The powder was stored at -20°C and was left resting at room temperature the night before the test. Differential scanning calorimetry (DSC1 Mettler Toledo®, Indium calibrated) was used to assess thermal stability degeneration of bone collagen. Twenty mL aluminium pans with flat bases were filled with ~10 mg of powder and the weight was recorded using a microbalance (Sartorius Genius ME235), while an empty crucible was used as a reference. The experiment was performed by steadily increasing the temperature from 25°C to 550°C at a rate of 10°C/min. The output curve was normalised and analysed with Stare V 10.00 software. This showed a first endothermic peak between 50°C and 120°C, which relates to the well-known collagen thermal shrinkage phenomenon[34] and a second exothermic peak between 200°C and 500°C that results from the combustion of the organic matrix[40–41]. For both episodes, enthalpy between a fixed temperature range, 30°C to 140°C and 200°C to 540°C, was calculated through integration and called respectively LΔH and CΔH. Furthermore, for each episode onset (LOnset and COnset), peak (LPeak and CPeak) and endset (LEndset and CEndset) was recorded. Finally, through a derivative the point of maximum steepness was detected as symptomatic of the collagen stability threshold (DerPeak1, DerPeak2) and for the combustion of the collagenous matrix (DerPeak3) [34,40,41].

### Thermo-gravimetric analysis

Gravimetric analysis (TGA 50 Mettler Toledo®, Curie Point calibrated) was applied to a sample of bone powder in the same conditions. A slightly bigger crucible (40 mL) was filled with ~20 mg of milled bone and the test was performed as previously described with a hold of the temperature at 550°C for the 10 final minutes in order to obtain ash weight. Quantitative investigation of the two main (percentage) weight losses with respect to final weight was recorded (Ash%) and that was carried out in Stare V 10.00 software by using horizontal tangents on the normalised curve. The first episode is believed to correspond to the complete dehydration of the bone (W%) while the second represents the combustion of the organic matrix (Or%).

### Statistical analysis

Statistical significance was set at P≤0.05 for both correlation values between the 23 parameters which we empirically measured and for the multifactorial regression analysis to predict biological age of the sample. One-way ANOVA was used to check for sex or ethnicity differences and once it was established that there were none the whole cohort of 24 donors was used for the subsequent analysis. The analysis consisted of producing a number of equations, through stepwise regressions, with consideration taken for the required degree of accuracy, time to complete the round of tests and resource availability. The entire statistical analysis was performed in Minitab v.17 and SPSS v.22.

## Results

A total of 28 physical parameters of interstitial bone (In) and osteons (On) were tested in ribs from 24 donors. Table 2 shows a list of the parameters, abbreviations, units, descriptive statistics and a list of experimental methods for data acquisition.

**Table 2:** List of the 28 physicochemical and histomorphometric parameters: Abbreviations, units, descriptive statistics and list of experimental methods for data acquisition.

Parameter	Abreviation	Units	Method	Mean	Median	SD
% optical porosity	%Po.Ar*	%	TLM/ImageJ	**11.07**	**11.77**	**5.15**
Indentation nanohardness for osteons	^**On**^H*	Vickers	Nanoindentation	**56.88**	**56.62**	**7.53**
Nanoindentation modulus (for Poisson’s ratio ν = 0.3) for osteons	^On^E_IT_	GPa	Nanoindentation	19.1	18.8	2.14
% indentation creep at hold (contact load) for osteons	^On^C_IT_	%	Nanoindentation	5.74	5.74	0.81
Elastic work % over the total (elastic + plastic) indentation energy for osteons	^On^η_IT_	%	Nanoindentation	20.19	20.67	1.8
Indentation nanohardness for interstitial bone	^It^H	Vickers	Nanoindentation	60.38	59.49	6.99
Nanoindentation modulus (for Poisson’s ratio ν = 0.3) for interstitial bone	^It^E_IT_	GPa	Nanoindentation	20.16	20.48	1.92
% indentation creep at hold (contact load) for interstitial bone	^It^C_IT_	%	Nanoindentation	5.87	5.84	0.7
Elastic work % over the total (elastic + plastic) indentation energy for intersitial bone	^It^η_IT_	%	Nanoindentation	20.87	20.74	1.87
Indentation microhardness of osteons	^On^HV	Vickers	Nanoindentation	30.84	30.44	4.29
Indentation microhardness of interstitial bone	^It^HV	Vickers	Nanoindentation	36.59	36.3	4.27
Pycnometry derived density	Dn_pyc_	g/cm^3^	Pycnometer	2.04	2.04	0.06
Numerical microcracks density	Cr.Dn*	n°/mm^2^	Pycnometer	**4.65**	**3.68**	**3.03**
Length density of microcracks	Cr.S.Dn*	mm/mm^2^	Pycnometer	**214.14**	**191.19**	**132.68**
Onset value of the endothermic episode	L_onset_	°C	DSC	53.21	54.44	6.4
Peak value of the endothermic episode	**L**_peak_*	°C	DSC	**96.2**	**96.83**	**4.21**
Endset value of the endothermic episode	L_endset_	°C	DSC	142.83	148.95	16.06
Enthalpy measurement of the endothermic episode	LΔH	J/g	DSC	120.77	112.26	22.97
Onset value of the exothermic episode	C_onset_	°C	DSC	289.28	289.51	3.1
Peak value of the exothermic episode	C_peak_	°C	DSC	353.11	355.94	19.69
Endset value of the exothermic episode	C_endset_	°C	DSC	41934	416.86	9.64
Enthalpy measurement of the exothermic episode	CΔH	J/g	DSC	3179.8	3265.5	637.47
Derivative defined value of the onset of the first endothermic episode	DerPeak1	°C	DSC	75.28	74.71	5.86
Derivative defined value of the endset peak of the first endothermic episode	DerPeak2	°C	DSC	119.67	120.19	6.47
Derivative defined value of the onset of the exothermic episode	DerPeak3*	°C	DSC	**325.57**	**323.96**	**6.58**
% value of water loss during TGA analysis	W_%_	%	TGA	8.21	8.32	0.71
% value of organic loss during TGA analysis	Or_%_	%	TGA	18.01	17.88	1.1
% Value of the final weight	Ash_%_	%	TGA	73.78	73.73	1.19

TLM = Transmitted Light Microscope, DSC = Differential Scanning Calorimeter, TGA = Thermo-Gravimetric Analysis, ImageJ = Image processing software. Significant (p<0.05) single correlations with age are shown with a * and in bold symbols.

Six parameters (%Po.Ar, ^On^H, Cr.Dn, Cr.S.Dn, Lpeak and DerPeak3) correlated singularly and significantly with age. The third derivative peak (DerPeak3) decreased, indicating a clear degeneration of the quality of the organic bone matrix and its amount in the bone tissue. The nanohardness of the osteonal area (^On^H) also decreased with age and showed a strong correlation (p = 0.029) and this change is linked in literature to the effect of age on bone plasticity[18,39]. Finally, optical porosity (%Po.Ar) drastically increased as a consequence of the physiological loss in bone mass that occurs throughout life. Although the study did not aim to investigate this relationship and did not perform any micromechanical test, no significant correlations were seen between numerical or crack length density and the nanoindentation parameters with age.

Significant positive correlation was observed between porosity and the percentage of organic loss during TGA analysis (Or_%_). The relationship between the parameters of differential scanning calorimetry (DSC) and those of the gravimetric analysis (TGA) denote a clear correlation between collagen integrity and amount of organic matrix present in the bone, which is in agreement with other studies[20]. Finally, the microhardness of the interstitial bone areas (^In^HV) appeared to be affected by the amount of organic matrix of the tissue that is present, which is expected.

### Stepwise regressions

Bone physicochemical characteristics not only have a direct influence on the mechanical integrity of the bone tissue but also carry useful information in order to investigate chronological age of human bone material. As shown in Table 2, only six parameters demonstrated significant correlations with age when singularly considered. This could be due to the limited variation of each parameter and its variance of the mean with regard to age for each individual [18,22]. Multifactorial stepwise analysis exhibited the predictive potential of different combinations of factors, as shown in Table 3. Different sets of variables were considered with forensic application and practice in mind and also bootstrapping methods were employed based on 1000 bootstraps.

**Table 3:** Stepwise and direct regression analysis produced 6 prominent equations as being most accurate.

*Equation*	*E1*	*E2*	*E3*	*E4*	*E5*	*E6*	
**Constant**	**-23.106**	**40.31**	**37.452**	**172.852**	**-13.599**	**-77.829**	***instrument***
**%Po.Ar**	**1.603**	**1.485**	**2.456**	**2.038**	**2.329**	**2.36**	*Optical*
P-Value	<0.001	<0.001	<0.001	<0.001	<0.001	<0.001	*microscope*
**Cr.Dn**	**3.021**	2.072					*Fluorescent*
*P-Value*	*0*.*03*	<0.001					*microscope*
**Cr.S.Dn**	***-0*.*035***						
*P-Value*	*0*.*076*						
**Dnpyc**						**45.79**	*Pycnometer*
*P-Value*						*0*.*16*	
^**On**^**C**_**IT**_	**4.435**						*Nanoindentation*
*P-Value*	*0*.*005*						
^**It**^**C**_**IT**_			**3.308**		**5.047**		
*P-Value*			0.103		*0*.*039*		
^**It**^**H**		***-0*.*407***					
*P-Value*		0.004					
^**On**^**HV**	***0*.*214***						
*P-Value*	*0*.*294*						
^**It**^**η**_**IT**_			**-1.724**				
*P-Value*			0.066				
**DerPeak1**	**-0.476**		**-0.667**				*DSC*
*P-Value*	*0*.*034*		0.044				
**DerPeak3**				**-0.373**			
*P-Value*				*0*.*066*			
**L**_**Onset**_	**0.802**		**0.813**				
*P-Value*	*0*.*001*		0.004				
**LPeak**				**0.743**			
*P-Value*				*0*.*092*			
**C**_**Endset**_				**-0.248**			
*P-Value*				*0*.*09*			
R^2^	0.949	0.912	0.902	0.848	0.82	0.81	
R^2^_adj_	0.927	0.899	0.875	0.816	0.804	0.792	

### Unrestricted parameter selection

Firstly, equations were sought by considering all 28 available parameters. In forensic terms this would be a situation in which time and resources were unlimited and the final goal was to reach the maximum accuracy and reliability so as to meet the increasing standards required for the admission of expert witness testimony. The best performing model by stepwise regression was Equation 1 (E1, Table 2) with a R^2^ = 0.949 (R_adj_^2^ adjusted for the degrees of freedom 0.927) showing good general accuracy; the maximum residual error of 2.88 years was observed for a 58-year-old individual (A8). The average residual error is 2.14±0.40 (SD). When bootstrapping is applied, the error becomes 2.77±1.43 (SD). E1 was created without taking into consideration time or resources availability; in fact, it required a two-week long preparation and the use of DSC, nanoindentation and fluorescence microscopy. E2 is the next stepwise regression and is based on just three variables (%Por.Ar, ^It^C_IT_ and Cr.Dn) produced by using the same instruments. The model shows R_adj_^2^ 0.912. The average residual error after bootstrapping is 2.75±1.47 (SD) and all coefficients present statistical significance.

### Restricted parameter selection

**Fig 4:**
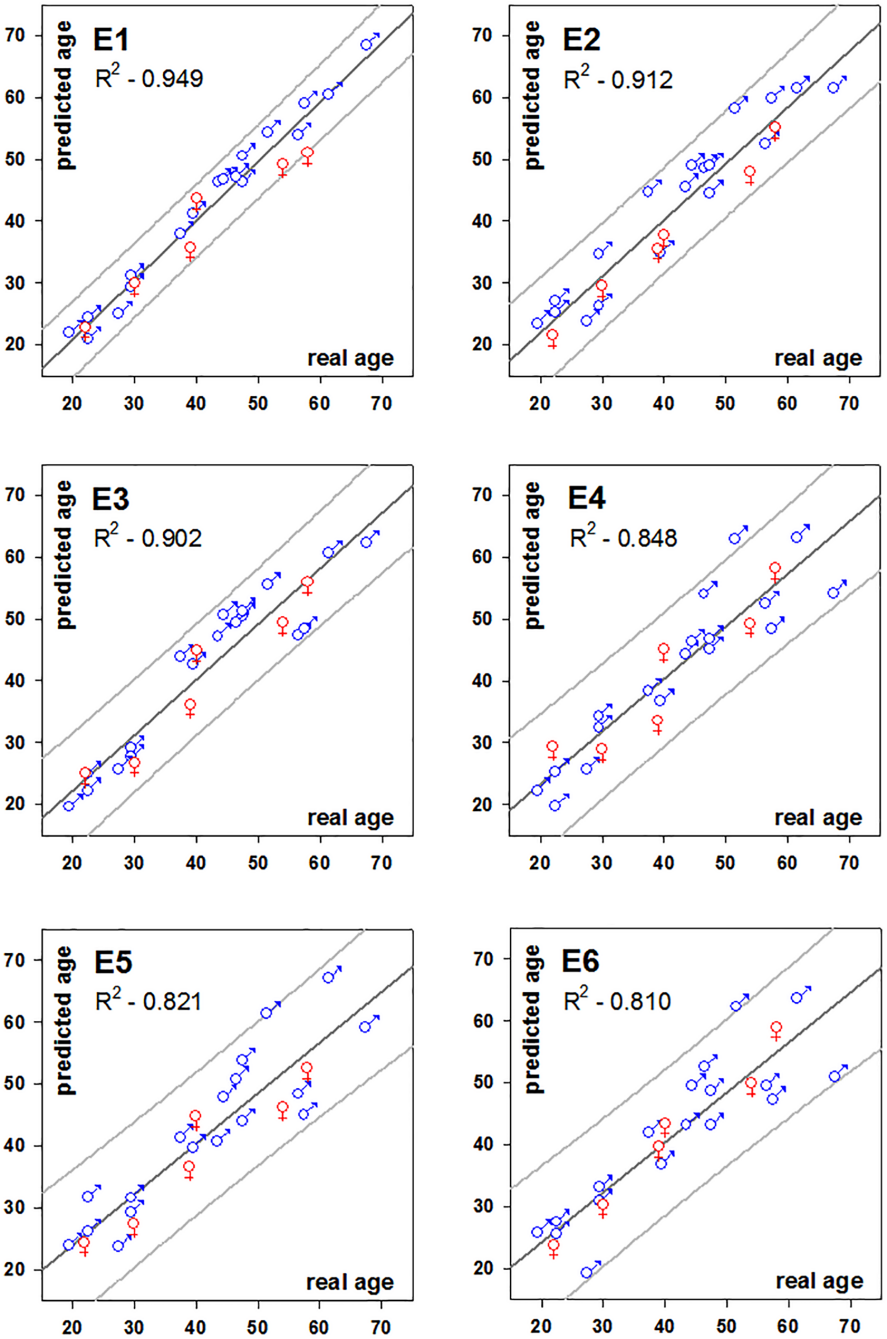
Plot of real age vs. predicted age for E1-E6. (Regression line with the 95% prediction interval for the data).

In further analysis we applied stepwise regression by using two specific subsets (combinations) of parameters in order to address practical problems that can occur in forensic context, such as limitations in time, technical resources, or available bone material. Equation 3 was derived by using the parameters produced by a DSC instrument, a Nanoindenter and the open source software ImageJ. The result was an equation (R^2^ = 0.902, R_adj_^2^ = 0.875) which was produced entirely by using bench top machines, and the entire experimental analysis can be conducted in 36 hours. For this predictive equation the residual errors ranged between 0.30 and 9.26 years. Certain other combinations were: E4 with four variables (%Po.Ar, Der Peak3, LPeak and C_Endset_) an R^2^ = 0.848 (R_adj_^2^ = 0.816); and E5 which involves the use of just one instrument a nanoindenter equipped with a microscope. E5 uses optical porosity (%Po.Ar) and the indentation creep (^In^CIT) values for the interstitial bone areas with R^2^ = 0.820 (R_adj_^2^ = 0.804). Although it requires just one instrument the preparation time may not be rapid enough for use in urgent legal cases. Finally, E6 was produced which requires the use of helium pycnometer and optical porosity (%Po.Ar). E6 could potentially be produced within 24 hours from the collection of samples. All six equations are shown in Fig 4.

### Cross-validation results

The previous models consider the whole cohort of samples and provide in essence the maximum possible prediction power of the approach we have implemented. In reality any unknown sample will be other than the samples which produced the calibration relationship. To simulate this, we applied a leave-one-out method where it turns we kept one sample out and produced the analysis from the other 23 samples. This was done 24 times x 6 predictive equations (6 sets of parameters). As expected cross-validation reduced the accuracy for E1 now having a mean average error of 4.41±2.58(SD) with errors in the range [0.28–9.42]yrs and highest error for a 39-year-old individual (Table 4).

**Table 4:** The age estimates for each subject when considered as unknown according to equations developed for the remaining 23 subjects (leave-one-out cross validation).

N	Age	E1	E2	E3	E4	E5	E6	Pathology
A1	45	46.57	49.77	52.49	46.34	48.66	50.65	NO Pathology
A2	39	29.58	34.46	33.72	30.69	35.73	38.98	Generalised atherosclerosis
A6	38	37.72	48.99	48.99	39.54	42.28	43.03	NO Pathology
A7	58	50.81	54.11	54.95	57.55	51.18	58.51	NO Pathology
A8	58	66.67	63.65	47.81	46.93	45.44	47.13	Coronary atherosclerosis, Valvular hypertension
A9	30	33.15	35.6	29.56	32.92	29.68	33.83	NO Pathology
A11	29	32.77	28.77	25.7	28.41	26.47	29.71	NO Pathology
A12	48	45.19	44.86	51.69	45.67	44.34	43.41	NO Pathology
A13	57	50.2	51.8	47.12	50.21	48.65	49.33	Coronary atherosclerosis
A17	54	45.66	47.02	48.11	47.84	45.25	48.66	Myocarditis, pulmonary oedema
A19	44	46.85	46.47	50.5	44.98	41.02	43.61	NO Pathology
A20	30	29.48	26.3	27.22	34.61	32.27	31.33	NO Pathology
C2	68	66.69	60.78	61.44	51.89	58.13	47.2	Hypertension, Coronary atherosclerosis,Progressive Supranuclear Palsy
C3	28	23.54	23.4	25.72	26.02	23.37	17.5	NO Pathology
C4	40	40.62	35.09	43.61	36.55	40.26	64.75	NO Pathology
C5	22	25.3	20.67	24.92	30.79	23.97	23.38	NO Pathology
C6	40	46.58	36.75	44.7	44.12	44.34	42.98	NO Pathology
C7	23	25.51	28.1	25.85	26.14	27.03	26.42	NO Pathology
C8	20	25	25.27	20.14	25.84	28.73	28.83	NO Pathology
C9	62	62.53	62.03	60.6	63.56	70.06	37.25	Alcohol abuse, smoking
C10	47	47.64	49.74	51	54.84	52.41	54.21	NO Pathology
C12	48	50.09	49.74	52.51	45.13	55.11	49.39	Fatty liver
C13	52	60.16	61.43	60.61	65.41	67.27	66.31	Hypertension
C22	23	20.22	26.13	22.4	13.69	34.46	28.49	NO Pathology
**<RE>**		4.41	3.89	4.65	5.23	5.86	6.08	
**SD**		2.58	2.93	3.08	4.23	3.88	4.54	

<RE>: mean absolute error of the estimation; SD: standard deviation

**Fig 5:**
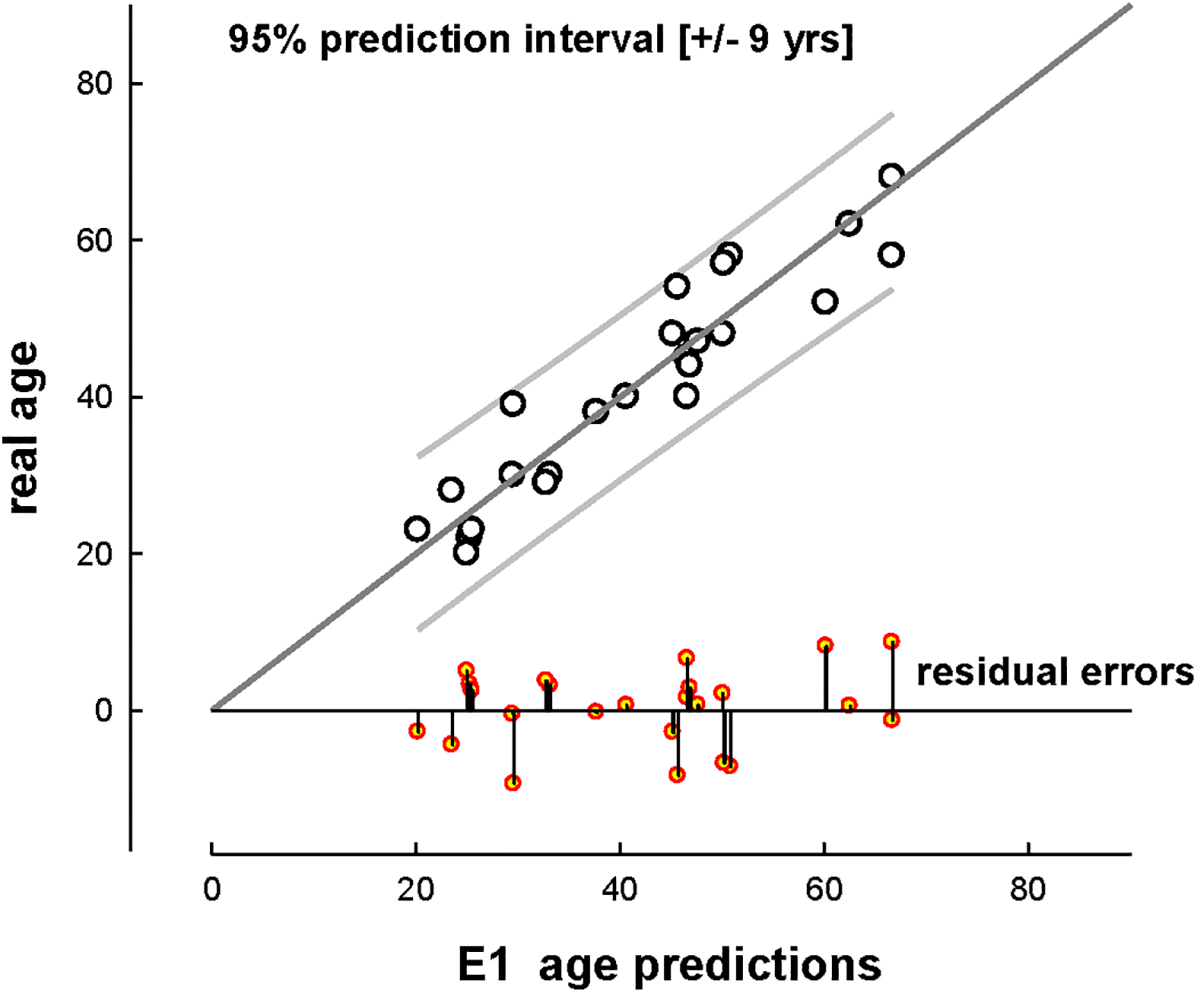
Plot of real age vs. predicted age for E1 in the cross-validation. Line of 1:1 equality with the 95% prediction interval for the data. Residual errors are shown on the x-axis for each donor (+ for overestimation;—for underestimated values).

Fig 5 shows the performance of E1 for the cross-validation study. The least performing E6, in cross-validation, showed mean residual error of 6.08 +/- 4.54 (SD) and the ‘worst’ single result (a residual age error of 20 years) was noted for a 52-year-old individual (C13) who suffered from hypertension.

## Discussion

Bone matrix undergoes de-/re-generation throughout life. Some changes such as bone mass loss and increase in porosity are easily quantifiable. Otherchanges that are of a more qualitative nature are caused by physicochemical alterations that affect both the mineral and the organic matrix[22]. All of these modifications can be quantified to a greater or lesser degree and can be correlated with the age of an individual. However, the rate of change decreases and is more difficult to detect in individuals who have already reached skeletal maturity (over 35 years old)[18]. It is believed that ribs are far less prone to remodelling from biomechanical stress compared to the femur[24–25, 42–43]; hence, they are the target of many histomorphometric studies for age-at-death estimation[4,5,11]. It is, however, well acknowledged that ribs are metabolically active and subject to many hormonal changes which also affect remodelling rates[43]. A few studies have focused on the material properties of the ribs in relation to age and fracture risk[44–46]. To date though, there is not a lot of information about nano-mechanical properties of the rib in relation to age. The current work attempts to explore the value of nano-material properties of the rib in forensic age estimation.

According to our results, several parameters exhibited significant correlations (P<0.001) with age thus making them potentially efficient age predictors. In addition, age estimates were fairly accurate for all age ranges, which is a significant advantage as compared to other age estimation methods based on ribs[4–5,8,11]. Lastly, preliminary observations suggest no influence of sex and population, which would make it ideal for global applicability. It is clear that further investigation should employ a large and well balanced sample in terms of age, sex and population to have a better understanding of the effect of these demographic features on the assessment and obtain a substantial understanding of the applicability in forensic setting.

In the present approach we employed a range of biomechanical and histomorphometric techniques that were listed in the methodology recently proposed by Zioupos et al.[18]. In addition, we also employed a microscopic examination of the bone matrix for the presence of in-vivo fatigue microdamage, a well-known detectable feature for ribs [33]. It is commonly accepted that the cyclic loading, which bone undergoes throughout life, results in the formation of microcracks at the microscopic level on both cortical and trabecular bone. What has been investigated here is the effect of cyclic loading on the mechanical quality of the cortical bone of the 4th rib and its quantitative changes with age. Decreases in toughness, strength and stiffness have been conclusively shown to correlate with the accumulation of micro-damage in the bone matrix[23,30,32,47]. Other physicochemical properties (such as mineral content, porosity and bone density) also relate to the accumulation of these cracks either as a cause or as an effect[42].

This study proposed six different regression equations for age estimation in the forensic context, according to the available time, specialised equipment and expertise. The best equation in terms of accuracy was found to be E1, which was produced from the entire heterogeneous cohort of parameters and presented a coefficient of determination R^2^ = 0.949; the maximum observed residual (error) was 5.15 years for a 58-year-old individual. When time and resources are limited, we recommend the use of E5, which can be completed in only 24 hours with the use of a nanoindenter and the open source software ImageJ. E5 had a coefficient of determination R^2^ = 0.830 and exhibited average residual error of 4.70±0.33 (SD), with a maximum of 12.57 years in one case. To put this into the forensic context and practice we have collated information for other laboratory-based age estimation methods[6,7,13,48–53] and age estimation methods based on the rib[4,5,8,11,54–57] from the literature including the present one. Comparing R^2^, which is the proportion of the variance in age that is explained by the regression model in each case and the mean residual error, E1 ranks third (see Table 5), making this, to our knowledge, the most accurate laboratory-based age estimation methods for ribs.

**Table 5:** Main laboratory- and skills-based methods for ribs in the literature.

**Reference**	**Method**	**R**^**2**^	**<RE>**	**SDE**
**E1 (present study)**	Rib	0.949	2.14	0.40
**Zioupos et al. (2014)**	femur	0.997	0.6	0.31
**Obert et al. (2013)**	skull	-	18	-
**Sakuma et al. (2012)**	teeth	0.87–0.96	7.4–3.9	-
**Schmitt et al. (2010)**	teeth	0.33	13.7	-
**Griffin et al. (2008)**	teeth	-	4.35	-
**Jankauskas et al. (2001)**	teeth	0.772	8.63	6.46
**Thomas et al. (2000)**	femur	0.574	9	-
**Martin de la Heras et al. (1999)**	teeth	0.47	14.3	-
**Ritz et al. (1996)**	skull	0.98	2.8	-
**Thompson and Galvin (1983)**	pelvis	0.798	6.33	-
**Verzeletti et al. (2010)**	Rib end morphology	0.357–0.935	1.958–6.278	-
**İşcan et al. (1984)**	Rib end morphology	0.76–0.85	0.72–1.21	-
**Stout and Paine (1992)**	Rib histomorphometry	0.721	3.9	-
**Stout et al. (1994)**	Rib histomorphometry		10.43	-
**Cho et al. (2002)**	Rib histomorphometry	0.569		
****				-
**Ohtani et al. (2002)**	Racemization of aspartic acid from rib cartilage	0.763	-	-
**Garamedi et al. (2011)**	Ossification of the first rib through radiographs	0.926	-	-

R^2^: coefficient of determination; <RE>: mean absolute residual error; SDE: standard deviation of absolute residual errors.

Despite the potential of the present study in assessing age at death, it remains a preliminary study and there are naturally limitations which need to be discussed. The sample comprised of individuals from two countries with similar dietary habits and customs. our results showed no effect of population specificity as the difference between the two population is negligible to obtain final results. Nevertheless, the composition of the sample did not allow us to explore potential sex-related differences or the effect of systematic pathologies affecting bone metabolism. Follow up research will investigate a larger and more balanced sample size in terms of sex, population and age ranges in order to reduce noise, taking into consideration dietary habits and metabolic diseases affecting bone, such as osteoporosis, that may interfere with the calibration of the method. Intra- and inter-observer error must be quantified in order to achieve standardisation of the technique, especially in relation to histomorphometric analysis. Furthermore, in order to understand better the relationship between microscopic and macroscopic structures in relation to the mechanical behaviour of bone, dynamic mechanical analysis (DMA) could also be tested. This can be performed by a bench top instrument and can be run efficiently for specimens of small size and mass. DMA would also provide variables that relates to the viscoelastic nature of bone[57] potentially adding to number of parameters used to predict age from skeletal remains. Finally, the post-mortem interval and diagenesis may play an important role in affecting bone quality (most likely through the organic matrix), and could put temporal limitations on the application of the present method. Restriction in time and resources availability did not allow for further investigation into this aspect, and remains a central issue that needs to be addressed.

## Conclusions

This study introduces a profoundly novel lab-based analytical method of ‘age at death’ estimation of skeletonised remains from the bone matrix properties of the human rib. For the development of the procedure we analysed the trends with age of as many as 28 biomechanical and material features of the human rib and created a mathematical model for age estimation that outstrips all previous published methods, whether these were phenomenological or analytical at the macroscopic or the microscopic level, or even chemical methods based on the analysis of proteins in soft and hard tissue. More importantly this approach can be easily replicated without need for the usual person centred high skilled forensic expertise. Its potential applicability ranges from unidentified skeletonised bodies to multiple victims of mass disasters or mass graves that are lacking identification and for which an accurate biological profile needs to be established. This makes the method relevant to chemists, biologists and medical experts that specialise in the field of forensics but it can also be relevant to any judicial personnel that deals with reliability and evidence admittance in the court of law. The present application was optimised for fresh (uncompromised by taphonomic conditions) remains, but the potential of the method is vast once the trends of the biomechanical variables are established for other environmental conditions and circumstances.
